# Incidence, causes and factors associated with torso injury in Cameroon: A community-based study

**DOI:** 10.7196/AJTCCM.2021.v27i3.161

**Published:** 2021-10-04

**Authors:** E Wepngong, S A Christie, R Oke, G Motwani, W Chendjou, K Azemafac, F M A Nour, D Dickson, R Dicker, C Juillard, A Chichom-Mefire

**Affiliations:** 1 Department of Surgery, Faculty of Health Sciences, University of Buea, Buea, Cameroon; 2 Center for Global Surgical Studies, Department of Surgery, University of California San Francisco, San Francisco, California, USA; 3 Program for the Advancement of Surgical Equity, Department of Surgery, University of California Los Angeles, California, USA

**Keywords:** torso injury, global surgery, injury, socioeconomic impact, road traffic injuries, cost of care, developing countries, Cameroon

## Abstract

**Background:**

Morbidity and mortality linked to injury has become an increasingly important public health concern worldwide, especially in developing countries. Despite the potentially severe nature of torso injury, little is known about the population-based epidemiology of torso injury in sub-Saharan Africa.

**Objectives:**

To determine the incidence, identify common mechanisms, and assess the socioeconomic consequences of torso injury in Cameroon.

**Methods:**

We performed a torso injury sub-analysis of a larger descriptive cross-sectional community-based study on injury epidemiology in the preceding 12 months in the Southwest region of Cameroon. Sampling was done using the three-stage cluster sampling technique. The differences between groups were evaluated using χ² and adjusted Wald tests.

**Results:**

We identified 39 cases of torso injuries out of 8 065 participants, providing a yearly incidence estimate of 488 (95% confidence interval (CI) 356 - 668) per 100 000 person-years. Road traffic injury was the most common mechanism of torso injury. The median (interquartile range (IQR)) cost of treatment for torso injury was USD58 (10 - 137), over four times the median (IQR) cost for non-torso injury at USD12 (3 - 43) (*p*=0.0004). About half of affected households (51%) reported being unable to afford necessities such as rent and food after injury v. 33% of households with members with non-torso injuries (*p*=0.018).

**Conclusion:**

Torso injuries have an incidence of 488/100 000 person-years, and road traffic injuries account for the majority of the injuries. Road traffic control measures and trauma care strengthening may reduce the impact of torso injuries and injuries in Cameroon.

## Background

Injury-related morbidity and mortality has become an increasingly important public health concern worldwide, especially in developing countries.^[1]^ The World Health Organization (WHO) lists injury as a leading cause of death, hospitalisation and long-term disability in the first four decades of life, with a mortality recorded every 6 seconds.^[2]^ Injury accounts for 9% of deaths worldwide and 12% of the global burden of disease with 90% of injury-related deaths occurring in developing countries.^[2]^ The burden of injury-related deaths is almost twice the number of fatalities associated with HIV/AIDS, tuberculosis and malaria combined.^[2]^

Torso injuries contribute significantly to the high burden of injury-related disease, and also the torso encloses delicate and vital organs that when injured, may become life-threatening and require prompt expert management, which is often not readily available in resource-poor settings.^[3–5]^ Moreover, rapid prehospital transport and specialised diagnostic techniques, which are associated with better outcomes, are limited in developing countries.^[4,5]^ Globally, chest injury accounts for 10% of trauma admissions and an estimated 20 - 25% of fatalities in trauma patients.^[6]^ The abdomen is the third most commonly injured region of the body, with 25% of patients requiring major surgery.^[7]^ About 9.3% of patients with blunt trauma usually end up with a pelvic fracture.^[8]^

Studies have been carried out to understand the global burden of injury as a whole but very few have focused on the critical torso region of the body, and even fewer have been carried out in developing countries despite the disproportionate impact of injuries in these settings.^[2]^ Moreover, the majority of available data in developing countries on injury is hospital-based, which inherently excludes injured subjects who do not present to the hospital despite having significant injury, either due to lack of access to care or preference for non-formal care, such as traditional bone setters.^[8,9]^ Community-level data are more representative than hospital-based data for informing the population-level burden of injury.

The objective of our study was to determine the incidence of torso injury in the community, identify common mechanisms of injury, and understand the burden posed on those affected in the Southwest region of Cameroon. Understanding the epidemiology, financial consequences, and therapeutic itineraries of torso injury can help inform appropriate healthcare resource allocation and interventions to improve access to care for those suffering from these high-acuity injuries. We hypothesise that road traffic injury is the most common mechanism of injury in the Southwest region of Cameroon, cost of care for torso injury is higher than non-torso injury, and the majority of those who sustain torso injury seek formal care compared with non-torso injuries.

## Methods

### Study design

We performed a torso (chest, abdominal, pelvic, upper and lower back) injury sub-analysis of a larger descriptive cross-sectional communitybased survey designed to estimate overall injury epidemiology and other surgical pathologies in the Southwest region of Cameroon.^[9]^

### Study setting

The study was carried out in the Southwest region of Cameroon, which has a mixed rural/urban distribution. The region has a total surface area of 25 410 km² and an estimated population of 1 575 224.^[10,11]^ The Southwest region is divided into 18 health districts, each of which is further divided into up to 12 distinct health areas.

### Study population

The study population consisted of all individuals residing in the Southwest region of Cameroon. Ineligible households were those in which only minors (<18 years) were present, non-consenting households, or those where no one was found at home after a minimum of two attempts.

### Sampling and sample size calculation 

Sampling was conducted using a three-stage clustered sampling framework.^[9]^ Using probability-proportionate-to-size sampling, in the first stage, nine health districts were selected and in the second stage, four health areas were selected per district. Population data estimates for each health district and area were provided by the Cameroon Ministry of Public Health and were used to generate weights for the study. Finally, in the third stage, a random starting point within each health area was selected using geolocation data to identify a starting household. Contiguous households were approached from that point until the target sample at each site was reached (*n*=200). A minimum sample size of 4 680 individuals was calculated to provide 78% power to detect an estimated 6% yearly injury incidence for the larger study, which was deliberately exceeded during data collection by ~50% at each site to account for multiple sub-analyses of relatively rare events.^[9]^

### Data collection and management

Over an 8-week period from January to March 2017, a structured questionnaire was administered to households after informed verbal consent was obtained by trained Cameroonian research assistants using a standard script. The family representative, a household member aged 18 years or older, provided injury information for all other household members on injury occurring in the 12-month period prior to survey administration.^[9]^

Data were obtained for torso injuries that occurred between January 2016 to January 2017. Non-torso injuries were injuries to other body regions excluding the torso. Injury was defined as any sudden bodily insult directly resulting in death or loss of routine activity by any family member for at least one day or that required medical care, regardless of whether such care was obtained.^[12]^ We collected sociodemographic characteristics such as age, sex, household location, type of cooking fuel used, highest level of education attained by a household member and home and agricultural land ownership, some of which served as markers of socioeconomic status.^[13]^ We also obtained data using the structured survey questionnaire on the total estimated cost of injury treatment and healthcare-seeking behaviour of the household such as whether formal care was sought or not and experience with formal care. The data were collected on paper surveys, then transferred into Research Electronic Data Capture (REDCap), a secure electronic database^[14]^ hosted on the University of California San Francisco server.

### Statistical analysis

Descriptive analyses were performed using frequencies, proportions, mean and standard errors (SEs) for continuous, normally distributed variables and medians with interquartile ranges (IQR) for nonparametric variables. Differences between groups were evaluated using χ² and adjusted Wald tests. Statistically, significance was set at p≤0.05. Population estimates were adjusted for clustering using the technique utilised in Demographic and Health Survey (DHS) estimates.^[11]^ Data analysis was done using STATA version 14 (STATA Corp., USA) and *svy* commands were used as appropriate to account for sampling weights and cluster survey design.

### Ethical consideration

Ethical approval was obtained from the Institutional Review board (IRB) of the University of Douala (ref. no. IEC-UD/694/10/2016/A) and University of California, San Francisco (ref. no. IRB#15-18424). Administrative authorisation was obtained from the Regional Delegation of Public Health, Southwest region.

## Results

### General characteristics of the study population

Of the 1 551 households approached for consent, 9.7% (*n*=151) were found to be ineligible while 7.3% (*n*=113) did not give consent. Therefore, a total of 1 287 (83.0%) households were included in our study, providing data for 8 065 subjects. The mean (SE) age of participants was 23.9 (18.2) years with a range of 0 to 115 years. Less than half of the participants (48.0%; *n*=3 865) were males (Table 1). Further details of the study population are described elsewhere.^[9]^

**Table 1:** Comparison of demographic and socioeconomic variables between individuals with torso, non-torso injuries and the rest of the population (*N*=8 065)*

	**Torso injury**	**Non-torso injury**	**No injury**	
**Variables**	**(*n*=39), *n*(%)^†^**	**(*n*=432), *n*(%)^†^**	**(*n*=7 594) *n*(%)^†^**	***p*-value**
**Age (years), mean (SE)**	28.7 (2.4)	27.3 (1.16)	23.9(0.3)	0.09
**Sex**				0.04
Male	19 (39.4)	267 (56.8)	3 560 (46.1)	
Female	19 (60.6)	161 (43.2)	3 969 (53.9)	
**Residence**				0.87
Urban	8 (34.6)	122 (39.7)	2 203 (39.3)	
Rural	31 (65.4)	305 (60.3)	5 284 (60.7)	
**Own agricultural land**	29 (68.0)	292 (63.4)	4 844 (56.8)	0.14
**Home ownership**				0.47
Own	23 (52.7)	256 (57.5)	4 788 (62.7)	
Rent	12 (36.5)	120 (33.5)	1 878 (29.7)	
Lives free	4 (10.8)	52 (9.0)	837 (7.6)	
**Cooking fuel**				
Wood	35 (88.8)	396 (89.5)	6 975 (88.7)	0.95
Charcoal	5 (22.8)	72 (22.4)	1 201 (22.0)	0.96
LPG	17 (48.3)	198 (54.9)	3 433 (52.5)	0.66
Kerosene	2 (10.9)	69 (16.7)	1 215 (19.6)	0.31
**Level of education**				0.49
None	0	9 (2.1)	148 (1.2)	
Primary	15 (38.6)	92 (16.9)	1 540 (16.9)	
Secondary	16 (37.2)	151 (34.9)	2 788 (36.4)	
Tertiary	7 (24.2)	170 (45.8)	2 956 (45.2)	
**Household possess cellphone**	36 (97.1)	404 (95.8)	7 075 (96.5)	0.67

SE = standard error

LPG = liquified petroleum gas

* Variables had missing data, hence the total *n* differs for each variable. Pearson’s χ² test or adjusted Wald test was used as appropriate. Percentages adjusted for the multi-cluster survey sampling method.

† Unless otherwise specified.

### Torso injuries

**Fig. 1:**
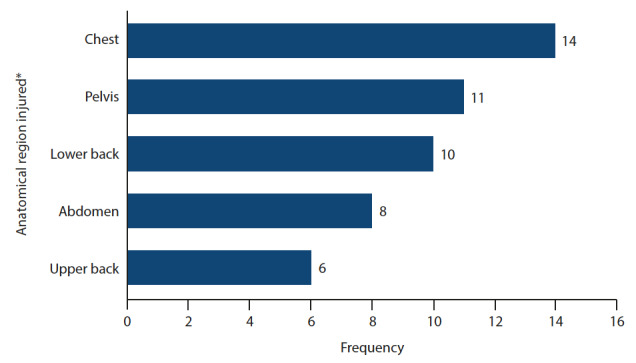
Torso injuries by anatomic distribution. * The anatomical regions injured were not mutually exclusive as each torso injury may affect more than one anatomic region.

Of 503 injuries reported,^[9]^ 39 torso injuries and 464 non-torso injuries occurred within the previous 12 months in the study cohort, giving an overall incidence of 488 (95% confidence interval (CI) 356 - 668) per 100 000 person-years. The chest (*n*=14) was found to be the most frequently injured torso region (Fig. 1).

The mean (SE) age of those who sustained torso injury was 28.7 (2.4) years and 65.4% (*n*=31) of them lived in a rural area (Table 1). Wood was the most used cooking fuel (88.8%; *n*=35) while over half (52.7%; *n*=23) of those with torso injuries owned homes. A higher proportion of subjects (56.8%; *n*=267) with non-torso injuries were males compared with 39.4% (*n*=19) and 46.1% (*n*=3560) for those with torso injuries and no injuries, respectively (*p*=0.04). All other sociodemographic variables for torso injuries were not statistically different from non-torso injuries and the uninjured study population (Table 1).

About a quarter of subjects with torso injury were crop farmers (26.0%; *n*=10) followed by students (21.6%; *n*=8). The top three activities resulting in torso injury were travel/transit (38.5%; *n*=15), work (30.8%; *n*=12) and leisure or playing (12.8%; *n*=5). A higher proportion (38.5%) of torso injured sustained the injury during travel/transit as compared with those with non-torso injuries (27.3%), while a slightly lower proportion (30.8%) were injured at work v. 33.9% of nontorso injuries (*p*=0.03).

**Fig. 2:**
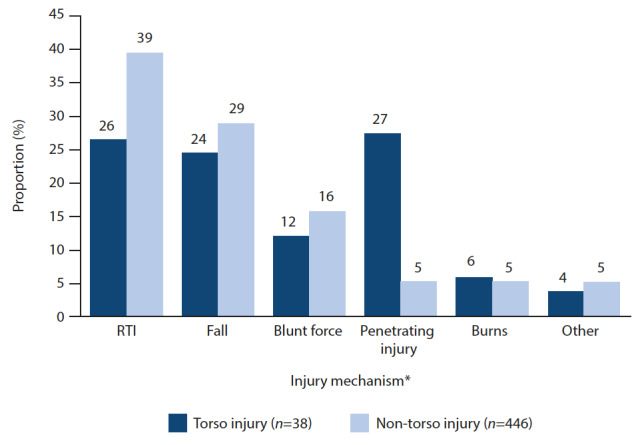
Mechanism of torso and non-torso injuries (n=484). RTI = road traffic injuri * Mechanism data only available for 484 of 503 reported injuries

Road traffic injury (RTI) was the most common mechanism of torso injury, accounting for 39.5% (*n*=15) injuries, followed by fall from height (29.0%; *n*=11) and blunt force (15.8%; *n*=6) (Fig. 2). Commercial motorbike riding accounted for 86.7% (*n*=13) of torso injuries sustained in a road traffic crash.

No subject with torso injury was reported to have died or stopped breathing on the day of the injury. However, 25.6% (*n*=10) were reported to have lost consciousness on the day of the injury v. 7.3% (*n*=34) of non-torso injuries (*p*=0.000), and 18.0% (*n*=7) were confused following the injury compared with 8.6% (*n*=40) of non-torso injuries (*p*=0.055).

**Care-seeking behaviour following the injury**

Most subjects with torso injury (82.1%; *n*=32) presented to formal medical care v. 61.9% (*n*=287) of non-torso injuries (*p*=0.012). Less than a quarter of the participants (18.0%; *n*=7) utilised home treatment or treatment by family, 7.7% (*n*=3) went to a bone setter or traditional healer, 5.1% (*n*=2) sought no form of care, and 15.4% (*n*=6) of participants utilised more than one care option following the injury

**Experience with formal care**

Of those who sought formal care, 81.3% (*n*=26) were satisfied with the care they received at the hospital, 9.4% (*n*=3) were dissatisfied, and 6.3% (*n*=2) were neutral. Among those with torso injuries who utilised formal care, 21.9% (*n*=7) reported having experienced long waiting times before being attended to, 25% (*n*=8) felt that they were treated disrespectfully by hospital staff, and 9.4% (*n*=3) were unable to pay for medical care and supplies.

**Economic impact of torso injury**

The median (IQR) cost of treatment for torso injury was USD58 (10 - 137). This was four times over the median (IQR) cost of treatment for non-torso injury at USD12 (3 - 43) (*p*=0.0004).

**Fig. 3:**
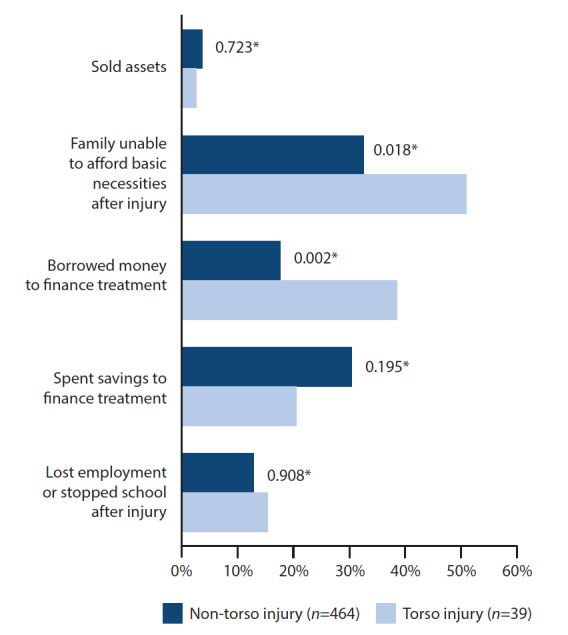
Economic consequences following torso injury v. non-torso injury (n=503).

About half of the households (51%; *n*=20) of torso-injured subjects reported being unable to afford necessities such as rent and food after injury v. 32.6% (*n*=143) of nontorso injuries (*p*=0.018), while 38.5% (*n*=15) of people with torso injuries borrowed money to finance treatment compared with 17.7% (*n*=82) of those with non-torso injuries (*p*=0.002) (Fig. 3).

A significantly higher proportion (76.9%; *n*=30) of those with torso injuries reported inability to carry out their primary occupation for at least a day, resulting in a loss of income v. 63.4% (*n*=286) of those with non-torso injuries (*p*=0.01), with a median (IQR) disability of 14 (7 - 90) days. About half of those with torso injuries (51.3%; *n*=20) had one family member (caretaker) cease their own income-generating activities for a median (IQR) of 14 (7 - 30) days owing to the injury, thereby affecting the household income.

## Discussion

In the present study, we sought to determine the incidence of torso injury from population-level data which is inherently more representative of injury burden, identify the most frequent mechanisms of injury and the burden it poses on affected households in the Southwest region of Cameroon. To the best of our knowledge, this is the first population-based study on torso injury providing previously unknown estimates conducted at the household level in Cameroon. We found an incidence of 488 torso injuries per 100 000 person-years. A third of torso injuries directly related to the chest, which is a significant contribution to the overall burden of diseases experienced by this population.

The mean (SE) age of participants (28.7 (2.4) years) with torso injury in our study is similar to the mean age of 29 years of the overall injured cohort in the main study^[9]^ and a mean (standard deviation) age of 28.6 (18.3) years of injured participants in an urban population-based study in India.^[15]^ This finding shows that the age group most affected with torso trauma are young individuals who constitute the workforce and also follows the trend of the overall injured age group. This highlights the potential economic impact of torso trauma as the most affected individuals are usually the breadwinners in their households.

Road traffic injuries were identified as the most common mechanism of injury in the present study, with most from commercial motorbikes. This finding is not surprising as commercial motorbikes are a means of transportation frequently used in developing countries and these regions have been experiencing a surge in the number of commercial motorbikes coupled with the poor road infrastructure.^[16,17]^ A survey conducted among motorbike riders and law enforcement agents in neighboring Nigeria found that poor road infrastructure, inadequate traffic control measures, and inadequately trained drivers contributed to road traffic collisions.^[18]^ Hence, implementation of preventive measures by regulators such as bike rider education, licensing and training, and improved road infrastructure may lead to significant reduction in the injury burden.

A large proportion of individuals who sustained torso injuries sought formal care, which deviates from what is often reported by studies on non-torso injuries in developing countries, where many people may prefer traditional bone setters or other non-formal care settings.^[19–21]^ This may imply that people perceive torso injuries to be more serious or prone to internal injuries that may not be readily apparent, which are better evaluated in a formal healthcare facility. Alternatively, as torso injuries may not include obvious findings of a broken bone, individuals with torso injuries may not readily seek non-formal care such as that provided by traditional bone setters. However, about a fifth of the study participants with torso injury did not seek formal care first, which highlights the need for further community sensitisation on the importance of seeking formal care as soon as possible following an injury. Although the majority of those who sought formal healthcare expressed satisfaction with the care they received, some still reported issues such as feeling disrespected by hospital staff, long waiting times before being seen, and inability to pay for hospital bills. These findings highlight the need for health system strengthening in developing countries aimed at quality improvement, enhanced healthcare financing and patient-centered care.

Torso injury poses a high economic burden on those affected, with the median cost of treatment being four times over the median cost for non-torso-injuries, further aggravating existing poverty. This finding may be related to a larger proportion of those with torso injuries seeking formal care, which is relatively more expensive than informal care.^[23]^ More than half of the households also reported inability to afford necessities such as food and rent following the injury, while some had to borrow funds to cover treatment. This finding is expected as up to 71% of the Cameroonian populace lives on <USD5.50 per day per capita.^[22]^ Torso injuries do not only infer financial hardship on those affected through the high cost of care, but they also causes disability days away from income-generating activities for both the injured person and caretakers. The significantly higher proportion of those with torso injuries who were unable to work and generate income contributes to perpetuating the prevalent poverty. Our study identified a higher number of disability days from work than a study conducted in India^[15]^ with a median (IQR) of 7 (4.0 -7.5) days for participants with moderate injuries and 2 (2 - 3) days for their caregivers. The higher number of disability days identified in our study may reflect the severity of torso injuries and the potentially longer recovery periods. This further contributes to the economic impact for the injured and their households. Adequate injury control measures are therefore needed to curb the socioeconomic impact of injuries in Cameroon.

### Study limitations

The number of torso injuries may have been underestimated, missed or misclassified, given that the cases were self-reported by participants and not verified by healthcare workers. There is also a possibility of recall bias, but this was minimised by limiting the period to the previous 12 months.^[23]^ The sociopolitical unrest in the Northwest and Southwest regions of Cameroon occurred during data collection and the study had to be paused for a period of about 3 weeks and two health districts were excluded to ensure safety of research personnel. Population-level data on torso injury are extremely limited so estimates were not available to guide sample size calculations. Although the original study design was intended for overall injury in the Southwest region, intentional oversampling was done to allow for sub-analysis of individual anatomic injuries such as torso injuries. 

## Conclusion

Torso injury contributes significantly to the overall incidence of injury in the Southwest region of Cameroon. It poses a high socioeconomic burden on individuals and households, with significantly increased cost of treatments and disability days away from daily income-generating activities when compared with non-torso injuries. The main mechanism of torso injury was road traffic injuries. Appropriate road traffic control measures and regulations by the government could reduce the impact of torso injuries and injuries in Cameroon as well as ameliorate the associated socioeconomic burden. A significant number of people still do not seek formal care following a torso injury for various reasons. This reiterates the need for health system strengthening and the meaningful use of resources by policymakers to improve access to quality healthcare. This could be achieved by investing in appropriate health infrastructure that will improve the working conditions of healthcare providers and the quality of healthcare rendered while reducing patient waiting time. Further studies should also be conducted to identify barriers and appropriate interventions that prevent individuals with injuries from seeking formal care. Finally, alleviating financial barriers to health by implementing a universal healthcare scheme will greatly improve the healthseeking behaviour of the population following an injury.
